# Regional One Health antimicrobial stewardship networks: a strategic framework for addressing antimicrobial resistance

**DOI:** 10.1017/ash.2026.10781

**Published:** 2026-07-03

**Authors:** Claudia Huebner, Steffen Flessa

**Affiliations:** Faculty of Law and Economics, Chair of Healthcare Management, University of Greifswald, Greifswald, Germany

## Abstract

Regional One Health antimicrobial stewardship networks require coordinated, adaptive strategies across sectors. This conceptual article combines strategic fit theory with the Ansoff matrix to identify key determinants of sustainable network performance and proposes strategic development pathways to strengthen governance, data integration, cross-sectoral collaboration and long-term network development.

## Objective

Antimicrobial resistance (AMR) represents a major public health challenge requiring coordinated responses across human, animal, and environmental health sectors.^1^ In this context, the One Health approach has become increasingly important for antimicrobial stewardship (AMS), particularly in regional healthcare settings where national strategies must be adapted to local epidemiological and organizational conditions.^2^

The outpatient sector poses particular challenges for AMS implementation.^3^ Antimicrobial prescribing is highly decentralized, governance structures are fragmented, and surveillance capacities are often limited. In addition, collaboration between human health, veterinary medicine, and environmental sectors remains insufficiently integrated.^4^ Although AMS is considered a key strategy for promoting responsible antibiotic use, cross-sectoral implementation remains limited, and multisectoral One Health approaches are still inconsistently applied, resulting in important gaps in both knowledge and practice.^5^

Regional AMS networks may help address these challenges by improving coordination, data integration, and cross-sectoral collaboration. Previous studies have identified important determinants of successful AMS implementation, including governance, communication, resources, surveillance systems, and stakeholder engagement.^6,7^ However, existing frameworks mainly focus on barriers and facilitators and provide limited guidance on how regional networks can strategically evolve under changing contextual conditions.

This article therefore proposes a theory-informed conceptual framework combining the strategic fit concept with the Ansoff matrix to support the development and sustainability of regional One Health AMS networks.

## Conceptual design

The manuscript is informed by systems theory, contingency theory and strategic management approaches, which conceptualize organizations as dynamic systems that continuously interact with changing external environmental conditions.^8^

The central analytical concept is strategic fit, which describes the alignment between external environmental demands and internal organizational capacities. In strategic management literature, this alignment is considered an important determinant of organizational sustainability and performance.^9^ Within regional AMS networks, external determinants include epidemiological resistance patterns, regulatory requirements, health policy priorities, financial framework conditions and technological developments. Internal determinants include governance structures, data infrastructure, intersectoral coordination mechanisms and cultural conditions.

To operationalize the strategic fit concept, the Ansoff matrix is applied as a heuristic framework for analyzing strategic development pathways in regional AMS networks. Originally developed to support organizational growth planning, the Ansoff concept distinguishes between different strategic options for adapting to changing environments and expanding organizational activities.^10^ It is used here not as a business performance model, but as a conceptual tool to structure adaptive development processes in complex health system settings. The interaction between external determinants and internal capacities determines the degree of strategic fit and informs strategic options.

**Figure 1.**
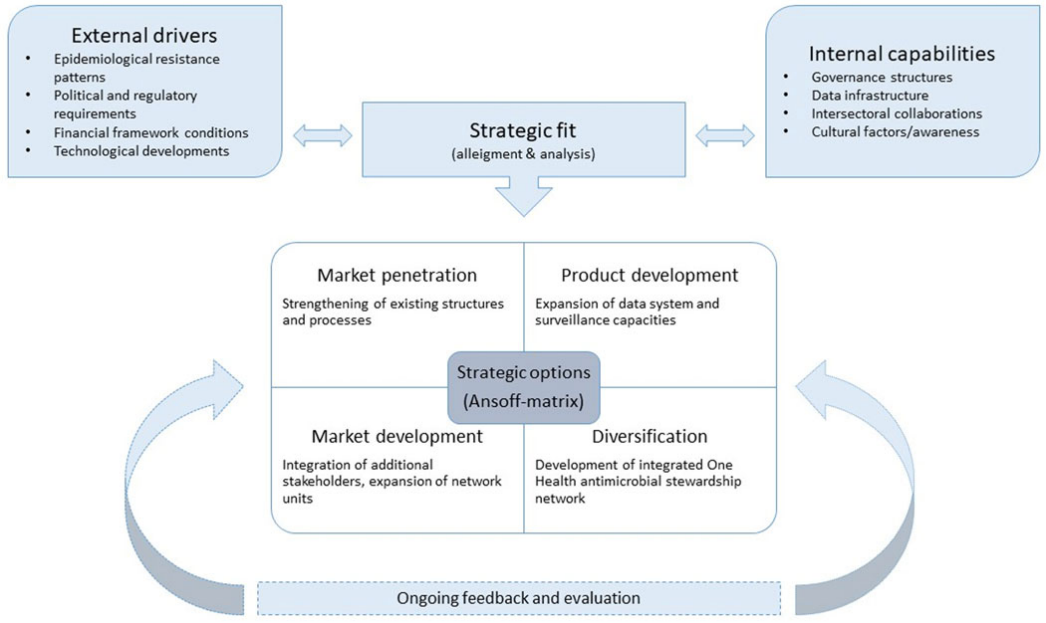
Conceptual framework illustrating the alignment between external determinants and internal capacities, the resulting strategic fit, and its translation into Ansoff-based strategic development pathways for regional One Health AMS networks.

This conceptual relationship is illustrated in Figure 1, which integrates external drivers, internal capabilities, strategic fit, and the resulting strategic options derived from the Ansoff matrix. In this study, the figure replaces a detailed textual repetition of the four strategic categories and provides a structured visual synthesis of the framework.

Overall, the framework links systems-level thinking with strategic management theory to explain how regional One Health AMS networks may adapt to changing contextual conditions through different strategic development pathways.

## Key determinants of sustainable One Health AMS networks

Several interdependent determinants appear to be central to the sustainability and effectiveness of regional One Health AMS networks. These include governance structures, resources and funding, data quality and interoperability, communication and trust, political and institutional support, a shared One Health culture, and monitoring and evaluation (Table 1).

**Table 1.** Key determinants of sustainable and effective regional One Health AMS networks

No.	Success factor	Description (brief)
(1)	Governance structures	Definition of responsibilities, coordination and decision-making processes within the network
(2)	Resources and funding	Availability of human, financial and time resources ensuring stability and continuity
(3)	Data quality and interoperability	Availability of high-quality, standardized and interoperable data for evidence-based decision-making
(4)	Communication and trust	Transparent communication and stable relationships enabling cross-sectoral cooperation
(5)	Political and institutional support	Regulatory and strategic frameworks ensuring legitimacy, embedding and stability
(6)	One Health culture	Shared values, understanding and readiness for change supporting cross-sectoral cooperation
(7)	Monitoring and evaluation	Continuous assessment and feedback enabling adaptive management and improvement of network activities

Together, these structural, technical, relational, and cultural determinants create an interconnected system that shapes network development, adaptive capacity, and long-term sustainability. Governance and resources provide the organizational foundation for coordinated action, while interoperable data systems, communication, and trust facilitate evidence-based decision-making and cross-sectoral collaboration. Political support, shared values, and continuous evaluation further strengthen network resilience and responsiveness to changing epidemiological and organizational conditions.

## Ansoff-based strategic development pathways

The Ansoff matrix provides a structured approach for translating these determinants into strategic development pathways for regional AMS networks (Figure 1). Market penetration focuses on strengthening existing structures and relationships, for example through improved stakeholder coordination, standardized prescribing feedback, or greater use of existing surveillance data. Product development aims to expand existing capacities and functions, such as interoperable surveillance dashboards, digital decision-support systems, and enhanced analytical capabilities.

Market development extends collaboration by involving additional sectors and institutions, including veterinary medicine, environmental agencies, public health authorities, and community healthcare providers. Diversification represents the creation of new cross-sectoral activities and structures, such as integrated One Health surveillance platforms or governance mechanisms linking human, veterinary, and environmental AMR monitoring.

## Illustrative application

The practical relevance of the framework can be illustrated using a hypothetical regional outpatient AMS network characterized by fragmented prescribing practices, limited data sharing and insufficient integration of veterinary and environmental stakeholders.

Within the market penetration dimension, improvements focus on strengthening existing coordination structures, for example through regular regional AMS meetings and the introduction of standardized prescribing feedback. Product development builds on current capacities by implementing interoperable surveillance dashboards and shared reporting systems that support more consistent and evidence-based decision-making across participating providers.

Market development extends collaboration by formally integrating veterinary and environmental actors into existing governance and coordination mechanisms. Finally, diversification represents a more transformative step through the establishment of an integrated regional One Health AMR platform that connects human, veterinary, and environmental surveillance systems. Overall, the example illustrates how the framework supports both incremental optimization and broader structural transformation of regional AMS networks.

## Discussion

The proposed framework integrates systems theory and strategic management perspectives to conceptualize how regional One Health AMS networks may respond to complex and changing external conditions. As illustrated in Figure 1, the interaction between external determinants and internal organizational capacities determines the degree of strategic fit, which in turn informs possible strategic development pathways based on the Ansoff matrix. The illustrative application further demonstrates how these pathways may be interpreted in a regional outpatient AMS context, ranging from incremental optimization to more transformative network expansion.

Compared with existing AMS and One Health implementation frameworks,^4,5^ which primarily identify barriers and facilitators, the proposed approach explicitly links these determinants to structured strategic options for network development over time. In this way, the framework provides a complementary perspective that emphasizes adaptive evolution rather than static implementation.

Importantly, the framework remains conceptual and does not claim empirical validation. The described outcomes should therefore be interpreted as potential implications of successful application rather than demonstrated effects. Future research is needed to empirically examine how the proposed determinants and strategic pathways operate across different regional and healthcare contexts, including through modeling and implementation studies.

Consequently, the framework should be viewed as a starting point for future research. Subsequent studies may build on this conceptual foundation through empirical investigation, implementation research, or formal modeling approaches to assess the relative importance of individual determinants and strategic pathways.

Overall, the framework offers a structured lens for understanding the development of regional AMS networks and may support both analytical reflection and future empirical investigation. In practice, successful implementation may be reflected in improved intersectoral data integration, more consistent antimicrobial prescribing, and strengthened cross-sectoral governance.

## Conclusion

AMR requires coordinated One Health responses. This article proposes a theory-informed framework combining strategic fit and the Ansoff matrix to guide the development of regional AMS networks. By linking key determinants with strategic development pathways, the framework provides a basis for future empirical research and implementation.
